# Estimating the Population Size of People Who Inject Drugs in 3 Cities in Zambia: Capture-Recapture, Successive Sampling, and Bayesian Consensus Estimation Methods

**DOI:** 10.2196/66551

**Published:** 2025-07-30

**Authors:** Lauren Parmley, Giles Reid, Joyce J Neal, Brave Hanunka, Leigh Tally, Lophina Chilukutu, Tepa Nkumbula, Chipili Mulemfwe, Lazarous Chelu, Ray Handema, John Mwale, Kennedy Mutale, Lloyd Mulenga, Anne F McIntyre, Neena M Philip, Hannah Chung, Maria Lahuerta

**Affiliations:** 1 Strategic Information Unit, ICAP at Columbia University, New York, NY, United States; 2 Bilateral Health Office, U.S. Agency for International Development/Southern Africa, 100 Totius Street, Pretoria, 0027, South Africa, 27 0764803906; 3 Survey Unit, ICAP at Columbia University, New York, NY, United States; 4 Division of Global HIV & TB, Global Health Center, U.S. Centers for Disease Control and Prevention, Phnom Penh, Cambodia; 5 Division of Global HIV & TB, Global Health Center, U.S. Centers for Disease Control and Prevention, Lusaka, Zambia; 6 ICAP at Columbia University, Lusaka, Zambia; 7 Department of Biomedical Sciences, Tropical Diseases Research Centre, Ndola, Zambia; 8 National HIV/AIDS/STI/TB Council, Lusaka, Zambia; 9 People Who Inject Drugs Sector, Key Populations Consortium, Lusaka, Zambia; 10 Ministry of Health, Lusaka, Zambia; 11 Division of Global HIV & TB, Global Health Center, U.S. Centers for Disease Control and Prevention, Atlanta, GA, United States; 12 Department of Epidemiology, Mailman School of Public Health, Columbia University, New York, NY, United States

**Keywords:** people who inject drugs, population size estimation, Zambia, PWID, HIV

## Abstract

**Background:**

Accurate population size estimates (PSE) of key populations—those disproportionately affected by HIV—are critical to forecast need and inform HIV prevention and treatment programs, though they can be difficult to ascertain due to low visibility of these groups. In Zambia, reliable estimates on the number of people who inject drugs are limited, inhibiting public health response.

**Objective:**

We sought to estimate the population size of people who inject drugs in 3 large cities in Zambia, assess how PSEs vary across different estimation methods, and explore the strengths and limitations of each approach.

**Methods:**

We applied 2-source capture-recapture (2S-CRC), 3-source capture-recapture (3S-CRC), and successive sampling population size estimation (SS-PSE) methods in Lusaka, Livingstone, and Ndola, Zambia. 3S-CRC methods included location-based 2S-CRC in combination with a respondent-driven sampling (RDS) survey. Data were collected from November 2021 to February 2022 and analyzed using a Bayesian nonparametric latent class model. SS-PSEs were produced using the RDS recruitment and network sizes. Kruskal tests and general linear models were used to examine sociodemographic and behavioral factors associated with being captured in 2S-CRC among RDS participants. Final city population estimates, incorporating 3S-CRC and SS-PSE with imputed visibility estimates, were generated using a Bayesian consensus estimator.

**Results:**

Bayesian consensus PSEs ranged between 0.5% and 1.8% of the adult male population and were below 1% of the total adult population in each city. Consensus estimates were highest in Lusaka (3700, 95% credible interval [CRI] 1500‐7500), followed by Ndola (2200, 95% CRI 1600‐2900) and Livingstone (1200, 95% CRI 900‐1,900). There was variability in estimates by method, with SS-PSE with imputed visibility generally providing the lowest estimates across cities, excluding Lusaka. Across methods, PSEs and uncertainty bounds (95% confidence interval [CI] or CRI depending on method) ranged from 1510 (95% CRI 1030‐2070) to 4350 (95% CI 1410‐18,890) in Lusaka, 360 (95% CI 290‐530) to 2620 (95% CRI 1510‐4680) in Livingstone, and 760 (95% CI 390‐3060) to 4030 (95% CRI 960‐5480) in Ndola. In all cities, fewer recaptures occurred in capture 3 (RDS) than with location sampling via 2S-CRC. Though results varied across cities, RDS participants captured through 2S-CRC differed from those captured solely through RDS in sociodemographic and behavioral risk factors, including housing, education, injection or needle sharing frequency, time since last injection, receipt of drug treatment, and experience with a peer educator in at least one city.

**Conclusions:**

This study used rigorous methods to produce PSEs in Zambia, and is the first to produce these for major geographies in the country. Through RDS, 3S-CRC reached people who inject drugs with distinct characteristics that were less accessible via location-based sampling (2S-CRC), yielding a PSE that may better reflect the population and informing the Bayesian consensus estimate. Findings from this study can guide program planning and future surveillance activities.

## Introduction

In Eastern and Southern Africa, 23% of new HIV infections are estimated to occur among key populations and their partners, roughly 1% of which are estimated to be among people who inject drugs based on available data [1,2]. Though population-level HIV estimates for people who inject drugs are limited, existing data indicate that people who inject drugs experience an HIV incidence rate 6.5 times higher than that of the general population in the region, with the proportion of new HIV infections and the incidence rate ratio slightly increasing from 2010 for this group [2]. Moreover, people who inject drugs endure other harms due to injection drug use, including infections from other blood-borne viruses such as hepatitis C virus (HCV) and hepatitis B virus (HBV), injury, and overdose [3].

Accurate population size estimates (PSEs) of key populations are critical to forecast need and inform HIV and HBV/HCV prevention, care, and treatment program planning, yet they can be difficult to ascertain due to the limited visibility of these groups. Difficulties in sampling key populations, including people who inject drugs, due to discrimination and criminalization, as well as nondisclosure of key populations in health care settings, can hinder PSEs using empiric methods. As a result, the availability and quality of PSEs for people who inject drugs vary. Only half of the countries in sub-Saharan Africa have injection drug use estimates—estimates of varied quality [3]. Although 1.4 million people have been estimated to inject drugs in the region, numbers may be as high as 3.1 million [3].

In Zambia, where punitive laws against drugs and injection equipment impede harm reduction services for people who inject drugs [4,5], estimates on the number of people who inject drugs are largely absent, limiting country-level planning and service delivery coverage estimates. While a formative qualitative assessment of people who use drugs was conducted in 4 cities in Zambia between 2013 and 2015 [6] and a size estimation study with people who inject drugs was conducted in 3 districts in Zambia in 2021 [7], prior methods used were of poor quality, included broad eligibility definitions related to HIV risk, or omitted major geographies in the country.

We sought to estimate the population size of people who inject drugs in 3 large cities in Zambia and document how PSEs vary across estimation methods for these groups. Multiple methods were used in the absence of a gold standard method for PSEs. We present findings, strengths, and limitations of each estimation method, including analyses that highlight method attributes, as well as our approach and results in generating a Bayesian consensus estimate for each city.

## Methods

### Study Setting

Data were collected from November 2021 to February 2022. Survey cities were selected to include 3 of the most populous cities in Zambia, which have a high number of people who use drugs according to PSEs from a prior study [6]. Each city has a different typology, with Livingstone serving as a major tourist destination and border town, the capital Lusaka serving as the economic and industrial hub of the country, and Ndola serving as a gateway to the Copperbelt province and mining region. Small area estimation techniques suggest the HIV prevalence among the general population in Livingstone, Lusaka, and Ndola is 14.9%, 16.5%, and 14.1%, respectively [8], higher than the national adult HIV prevalence of 9.8% [9].

### 2-Source Capture-Recapture and 3-Source Capture-Recapture: Captures 1 and 2

In each city, we implemented multiple source capture-recapture where people who inject drugs were “sampled” through 3 captures. This included the distribution of unique objects at places or “hotspots” where people who inject drugs congregate at 2 points in time (captures 1 and 2), followed by a biobehavioral survey (BBS; capture 3). Details and assumptions on capture-recapture methods have been described previously, including that capture-recapture relies on assumptions specific to population closure, capture independence, homogeneous capture probabilities, and clearly defined captures [10-12]. For captures 1 and 2, implemented a week apart, volunteers had a target sample size to distribute up to twice the number of unique objects as the target BBS sample size per capture in each city (target sample size and sample size calculations for the BBS are described further below). Distribution locations and appropriate distribution objects (black and white bracelets for captures 1 and 2, respectively) were identified through a formative assessment and through support from organizations who work with people who inject drugs. Volunteers were assigned to distribute objects at these locations as well as additional locations in the volunteer’s neighborhood or preferred hangout place. Distribution occurred over 3 days to limit the possibility of someone receiving objects from different volunteers. Each day of distribution, volunteers (13-27 per city and capture) received paper-based distribution logs to be completed during distribution, along with their allotted number of objects to distribute in their allocated daily catchment areas. Different volunteers were used for each capture to reduce dependence between captures, and any participating volunteer was excluded from the seed selection for capture 3. During each of the first 2 captures, volunteers approached both known and potential members of the people who inject drugs community and verified whether individuals had injected drugs within the past 3 months and were 16 years of age or older. After confirming eligibility (same as BBS eligibility below), volunteers inquired whether individuals had previously received the unique object and briefed them about the unique object distribution process and the upcoming survey. If individuals had not previously received the object in that capture, they were offered an object. People who inject drugs who accepted objects in capture 1 were encouraged to keep their object for 2 weeks and told that they may be asked about it in the future. In capture 2, volunteers verified if individuals had received the unique object distributed in capture 1, though they were not required to have it on them as long as they confirmed receiving it. Eligibility, object acceptance or refusal, prior receipt of object, date, time, and location of distribution were recorded on the log and later entered via tablet questionnaire. Any objects that were not distributed were returned to the survey team.

### 3-Source Capture-Recapture: Capture 3 (BBS)

Capture 3 comprised a BBS with survey methods aligned to the Biobehavioural Survey Guidelines for Populations at Risk for HIV [13]. Participants were recruited using respondent-driven sampling (RDS), a chain referral approach used to recruit populations for whom no sampling frame exists [14]. Individuals were eligible to participate in the BBS if they met the following criteria: self-reported drug injection for nonmedical purposes in the past 3 months; aged 16 years or older; lived in the surveyed city for the past 3 months; spoke English, Bemba, Kaonde, Lozi, Nyanja, or Tonga; willing to provide verbal informed consent; and in possession of a valid survey coupon (for coupon recipients). Seeds, well-connected people who inject drugs who met the eligibility criteria, were identified through a formative assessment and through support from organizations serving people who inject drugs. Seeds were purposively selected to ensure diversity across age, sex, education, area of residence, language, nationality, socioeconomic status, and key populations organization affiliation, and were instructed to recruit 3 of their peers via coupon. Peers who were eligible and participated in the BBS were provided 3 coupons and encouraged to recruit 3 of their peers, and so on.

After providing consent, tablet-based questionnaires were administered via interviewer and covered topics such as demographics, sexual and injection risk behaviors, knowledge, attitudes, and practices related to HIV, stigma and discrimination, and social and structural networks. Data validation and completion checks were preprogrammed to minimize data entry errors. To inform 3-source capture-recapture (3S-CRC), participants were asked about object acceptance or refusal and prior receipt of objects for both captures 1 and 2. Where participants did not physically possess the objects during the interview, they were asked to identify the objects from several images. Interviews lasted approximately 1 hour, after which participants who provided consent were tested for HIV, syphilis, HCV, and HBV.

Using the sample size calculator for survey-based viral load suppression in the Biobehavioural Survey Guidelines for Populations at Risk for HIV as is best practice for BBS [13] and assuming a design effect of 2 and a nonresponse rate of 5%, we calculated a sample size of 195 HIV-positive participants would produce a 2-sided 95% confidence interval (CI) ranging from 50% to 70% when viral load suppression among HIV-positive participants was 60%. To achieve a sample size of 195 HIV-positive participants per survey city, assuming an HIV prevalence of 25%, we estimated a sample size of 780 participants per survey city. After consultation with key population stakeholders in Zambia, it was determined that the target sample size of 780 people who inject drugs would not be achievable in each city, given the expected size of the population and the willingness of people who inject drugs to participate in a study. This sample was subsequently allocated across the 3 cities based on population size and recruitment feasibility. Each city’s target sample size was then increased slightly to improve the precision of city estimates and better assure statistical equilibrium and convergence (Lusaka: n=350, Livingstone: n=235, and Ndola: n=260).

### Successive Sampling Population Size Estimation

Successive sampling population size estimation (SS-PSE) methods [15] with and without imputed visibility were also used to estimate the population size of people who inject drugs in the survey cities. SS-PSE models the total number of persons in the target population using RDS data from the BBS and, as a result, relies on the inherent assumption that the social network of the population of interest is fully connected. It also assumes that the probability of recruitment for an individual is proportional to their network size, and that the network size is known accurately. SS-PSE methods use the self-reported individual network size question asked in the survey, combined with a prior estimate for the population size, and a Bayesian model to estimate the size of the target population [16]. SS-PSE with imputed visibility is an extension that introduces a measurement error model for network size, which helps account for misreporting and bias in self-reported network size [17].

### Bayesian Consensus Estimation

For published reports, technical and stakeholder consultation guided the statistical estimation of a single consensus estimate, which combined estimates from the separate PSE methods.

Consultation took place in 3 parts. First, the project technical staff reviewed and compiled prior information, then computed the PSE using each method. The results were discussed, and an initial decision was made to include only the SS-PSE with imputed visibility and 3S-CRC estimates, based on data quality and plausibility. To elicit feedback from the wider project and key population interest groups, a meeting was convened with the Zambia Key Populations Consortium, a network of key population members. Priors and confidence in each method were finalized for input into the Bayesian consensus estimates calculation (using the “Triangulator” app, further described below).

### Statistical Analysis

Data analysis for counts, proportions, and PSEs was conducted using R (version 4.0.5; R Foundation for Statistical Computing). PSEs and credible intervals (CRIs) for 2-source capture-recapture (2S-CRC) and 3S-CRC were derived using a Bayesian nonparametric latent-class model in the *shinyrecap* package [18]. Size estimation was based on the number of participants overlapping (or recaptured) across capture events. The Bayesian model assumes that there are several unobserved subgroups of the population which each have different capture probabilities for each capture event. Using RDS data, bivariate analysis was used to compare sociodemographic factors and drug injection practices by capture history to understand potential differences between those captured through 2S-CRC or the BBS alone.

SS-PSE was computed with the Gile estimator in the *sspse* package (version 0.6) using RDS recruitment histories and self-reported network sizes [19]; measurement errors were estimated with and without imputed visibility. There was very limited information on people who inject drugs in the study cities before our study, so prior populations for SS-PSE were based primarily on the percentage of the population who were estimated to be injection drug users based on a review of previous studies in sub-Saharan Africa [20]. Other evaluations of the people who inject drugs population in Zambia suggested that almost all people who inject drugs were male, so census estimates of the male aged 15‐64 years population for each city were used to compute priors based on an estimate of 0.6% of this population being people who inject drugs [7]. This resulted in initial estimates of 5000 for Lusaka, 1200 for Ndola, and 400 for Livingstone. The Livingstone prior estimate was increased to 800, because the study contacted more than 800 distinct people who inject drugs during the unique object distribution and the RDS recruitment. To account for the large prior uncertainty while constraining estimates to be plausible proportions of the general population in each city, we set the lower quartile for the prior at half of these estimates, and the upper quartile as twice these estimates. The prior estimates were reviewed and agreed with key population stakeholders and experts from the collaborating Zambia organizations before being input into the SS-PSE calculation.

Results from the RDS survey were produced using R (version 4.0.5, *RDS* package, version 0.9‐3) with bootstrap variance estimation of 95% CIs [21]. Using Taylor series variance estimation, SAS (version 9.4; SAS Institute Inc) was used for indicator validation and sensitivity analyses. Complete case analysis was used when data were missing, as missing data were minimal.

Final statistical computation of the consensus estimate was carried out using a Bayesian approach, which weights the available estimates and their variance by a “design confidence” weight based on judgment of the methodological reliability of each estimate [22]. Design confidence scores of 75 and 65 were assigned to the 3S-CRC estimates and SS-PSE with imputed visibility estimates, respectively. These confidence factors were set conservatively in collaboration with this study’s technical group to account for potentially unmet assumptions and to recognize the substantial divergence of the estimates from different methods from each other and our original priors. Priors for the consensus estimate were set for the lower and upper limits of the people who inject drugs population in each city. Lower bounds used the number of unique individuals captured in each city, and upper bounds were set at approximately 10% of the total city population to avoid unwanted downward influence on the estimates.

### Ethical Considerations

This activity was reviewed and approved by the United States Centers for Disease Control and Prevention, Columbia University Medical Center Institutional Review Board, Tropical Diseases Research Centre Ethics Review Committee, and the Zambia Ministry of Health National Health Research Authority. Permission and administrative approval from the Zambia Ministry of Health and the National Health Research Authority were obtained before data collection. Letters of support from the Ministry of Home Affairs (Police) and the Drug Enforcement Commission were obtained to ensure that researchers and people who inject drugs were not prosecuted during the survey’s period. Consent was neither required nor sought from people who inject drugs in captures 1 or 2, as aggregate data collected were not classified as human subjects research. To reduce the potential risk of breach of confidentiality, BBS participants provided verbal informed consent. BBS data collection sites were selected to ensure privacy protection, and all survey staff were trained in Good Clinical Practices and signed a confidentiality agreement. Participants received a maximum of K430 (approximately US $27) in cash for compensation of transportation costs, time, and peer referrals in the BBS. Nonidentifying codes were used to link participant questionnaire and biomarker data. Data included in this analysis were deidentified.

## Results

### 2S-CRC: Captures 1 and 2

In Livingstone, 426 and 432 individuals were approached in captures 1 and 2, respectively. In capture 1, a total of 415 (97%) individuals were eligible, and of those, 20 had previously received an object and been sampled in the capture (Table 1). Of those who had not previously been captured and were offered an object, 97% accepted, resulting in a total of 383 people who inject drugs sampled in capture 1. In capture 2, a total of 421 (97%) met study eligibility criteria, and of those, 7 had previously received an object and been sampled in capture 2. Overall, 411 people who inject drugs accepted an object and were sampled in capture 2. Of those sampled in capture 2, a total of 99 (24%) self-reported to having received an object in the first capture and been sampled in capture 1.

**Table 1.** Eligibility and object acceptance among people who inject drugs approached across 3-source capture-recapture methods by city, Zambia, 2021.

People who inject drugs approached	City								
	Livingstone			Lusaka			Ndola		
	Capture 1 (n=426), n (%)	Capture 2 (n=432), n (%)	Capture 3 (n=249), n (%)	Capture 1 (n=700), n (%)	Capture 2 (n=763), n (%)	Capture 3 (n=479), n (%)	Capture 1 (n=449), n (%)	Capture 2 (n=473), n (%)	Capture 3 (n=303), n (%)
Self-reported to be people who inject drugs^a^									
Yes	415 (97)	421 (97)	235 (94)	617 (88)	697 (91)	349 (73)	442 (98)	450 (95)	259 (85)
No	11 (3)	11 (3)	14 (6)	83 (12)	66 (9)	130 (27)	7 (2)	23 (5)	44 (15)
Already in capture^b^									
Yes	20 (5)	7 (2)	0 (0)	29 (5)	73 (10)	0	1 (0)	3 (1)	0 (0)
No	395 (95)	414 (98)	235 (100)	588 (95)	624 (90)	349 (100)	441 (100)	447 (99)	259 (100)
Object acceptance^c^									
Accepted	383 (97)	411 (99)	N/A^d^	548 (93)	597 (96)	N/A	428 (97)	432 (93)	N/A
Refused	12 (3)	3 (1)	N/A	40 (7)	27 (4)	N/A	13 (3)	15 (3)	N/A

a For capture 3, there were additional eligibility criteria, as noted above in the Methods section.

b Among those who self-reported to be people who inject drugs.

c Among those not captured in the current capture.

d N/A: not applicable.

In Lusaka, 700 and 763 individuals were approached in captures 1 and 2, respectively. In capture 1, a total of 617 (88%) individuals were eligible, and of those, 29 had previously received an object and been sampled in the capture. Of those who had not previously been captured and were offered an object, 93% accepted, resulting in a total of 548 people who inject drugs sampled in capture 1. In capture 2, a total of 697 (91%) met study eligibility criteria, and of those, 73 had previously received an object and been sampled in capture 2. Overall, 597 people who inject drugs accepted an object and were sampled in capture 2. Of those sampled in capture 2, a total of 209 (35%) self-reported to having received an object in the first capture and been sampled in capture 1.

In Ndola, 449 and 473 individuals were approached in captures 1 and 2, respectively. In capture 1, a total of 442 (98%) individuals were eligible, and of those, 1 had previously received an object and had been sampled in the same capture. Of those who had not previously been captured and were offered an object, 97% accepted, resulting in a total of 428 people who inject drugs sampled in capture 1. In capture 2, a total of 450 (95%) met study eligibility criteria, and of those, 3 had previously received an object and been sampled in capture 2. Overall, 432 people who inject drugs accepted an object and were sampled in capture 2. Of those sampled in capture 2, a total of 44 (10%) self-reported having received an object in the first capture and been sampled in capture 1.

PSEs generated using 2S-CRC were highest in Ndola (4030, 95% CRI 960‐5480), followed by Lusaka (1510, 95% CRI 1030‐2070) and Livingstone (1550, 95% CRI 870‐1930; Table 2).

**Table 2.** Population size estimates of people who inject drugs by city and estimation method, Zambia, 2021.

PSE^a^ method^e^	City		
	Livingstone PSE (95% CI/CRI^b^)	Lusaka PSE (95% CI/CRI)	Ndola PSE (95% CI/CRI)
Capture-recapture			
2-source capture-recapture	1550 (870-1930)	1510 (1030-2070)	4030 (960-5480)
3-source capture-recapture	2620 (1510-4680)	2320 (1690-10,740)	3350 (2820-3930)
SS-PSE^c^^,^^d^			
SS-PSE	450 (340‐810)	4350 (1410-18,890)	2010 (690-7400)
SS-PSE with imputed visibility	360 (290‐530)	2040 (660-30,410)	760 (390‐3060)

a PSE: population size estimation.

b CI: confidence interval; CRI: credible interval. Capture-recapture methods report CRIs while SS-PSE report CIs.

c SS-PSE: successive sampling population size estimation.

d SS-PSE represents medians.

e Estimates and CIs/CRIs are rounded to the nearest tens.

Across cities, the most common recruitment locations for captures 1 and 2 were bars, taverns, clubs, or shebeens (informal drinking establishments), as well as streets or parks in Lusaka and Livingstone. More than half of the people who inject drugs in Ndola were recruited from bars, taverns, clubs, or shebeens in both captures and recruitment from this location type increased nearly 2-fold between captures 1 and 2 in Lusaka and Livingstone. Across all cities, many people who inject drugs were recruited from “other” locations, which included free-text responses of homes (capture 1: 25%; capture 2: 5%) and markets or shops (capture 1: 11%; capture 2: 8%).

### 3S-CRC: Capture 3 (BBS)

Lusaka recruited the largest number of purposive seeds (n=12) to participate in the BBS, followed by Ndola (n=8), and Livingstone (n=7). In Livingstone, 249 individuals were screened, 235 were eligible, and 235 were enrolled in the BBS (Table 1). In Lusaka, 479 individuals were screened, 349 were eligible, and 349 were enrolled. In Ndola, 303 individuals were screened, 259 were eligible, and 259 were enrolled. Coupon return rates were highest in Ndola (54%), followed by Lusaka (52%) and Livingstone (48%).

The target population was predominantly male, with an estimated 83% (95% CI 73.9‐92.9) of people who inject drugs in Livingstone, 96% (95% CI 94.9‐98.0) of people who inject drugs in Lusaka, and 67% (95% CI 58.2‐75.6) of people who inject drugs in Ndola assigned male sex at birth. Median age (IQR) was 22 (20-28), 25 (22-29), and 27 (23-32) years for men in Livingstone, Lusaka, and Ndola, respectively, and 29 (23-34), 22 (21-24), and 29 (24-35) years for women in Livingstone, Lusaka, and Ndola, respectively. Most people who inject drugs completed secondary school education or higher (Livingstone: 56%, 95% CI 50.1‐63.0; Lusaka: 62%, 95% CI 56.7‐67.4; Ndola: 87%, 95% CI 82.6‐91.4) and were currently unemployed (Livingstone: 58%, 95% CI 51.4‐66.0; Lusaka: 78%, 95% CI 72.8‐83.4; Ndola: 45%, 95% CI 26.6‐36.0). All but 7 of those recruited across all 3 cities were born in Zambia.

**Figure 1.**
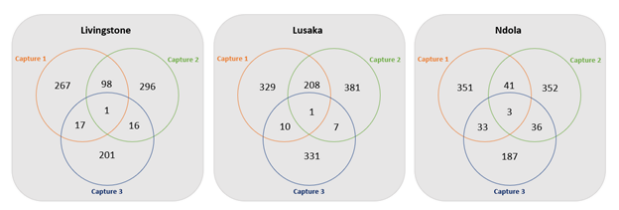
Venn diagram of total captures and recaptures of people who inject drugs from 3-source capture-recapture methods by city, Zambia, 2021.

Figure 1 provides overlaps or recaptures across all captures by city, which were used as inputs to generate PSEs using 3S-CRC. In all cities, fewer overlaps occurred with 3S-CRC than with 2S-CRC. PSEs generated using 3S-CRC were highest in Ndola (3350, 95% CRI 2820‐3930), followed by Livingstone (2620, 95% CRI 1510‐4680) and Lusaka (2320, 95% CRI 1690‐10,740; Table 2).

Bivariate analysis demonstrated differences in sociodemographic factors and drug injection practices among people who inject drugs by capture history (Table 3). In Livingstone, people who inject drugs participating in the BBS who were not captured via location sampling (2S-CRC) had a higher prevalence of homelessness, levels of education completed, and receipt of drug treatment, and differences in the frequency of injection in the last 6 months and of ever “blue toothing”—injecting oneself with a friend’s blood to get high—compared to BBS participants who were previously captured. In Lusaka, people who inject drugs participating in the BBS and who were not captured via location sampling had a higher prevalence of living in a home compared to other housing types, more frequent injection in the last 6 months, never using a shared needle, and had differences in levels of education completed, and time of last injection compared to BBS participants who were previously captured. In Ndola, people who inject drugs participating in the BBS who were not captured via location sampling had a higher prevalence of recent injection and never using a shared needle and a lower prevalence of ever speaking to a peer educator or outreach worker about HIV, levels of education completed, and ever blue toothing, and had differences in housing type compared to BBS participants who were previously captured.

**Table 3.** Bivariate analysis of sociodemographic factors and drug injection behaviors among people who inject drugs in the respondent-driven sampling biobehavioral survey by capture history and city, Zambia, 2021.

	City								
	Livingstone			Lusaka			Ndola		
	Previously captured^a^ (n=34), % (95% CI^b^)	Not previously captured (n=201), % (95% CI)	*P* value^c^	Previously captured^a^ (n=18), % (95% CI)	Not previously captured (n=331), % (95% CI)	*P* value^c^	Previously captured^a^ (n=72), % (95% CI)	Not previously captured (n=187), % (95% CI)	*P* value^c^
Age (years), median (IQR)	22 (18‐29)	23 (20‐30)	.01	30 (27‐32)	24 (21‐28)	.13	25 (22‐32)	28 (24‐34)	.11
Sex			.57			.44			.42
Male	78.5 (59.0‐97.9)	83.1 (74.9‐91.4)		93.7 (87.6‐99.8)	96.6 (94.5‐98.7)		62.1 (49.2‐76.0)	68.9 (59.1‐78.6)	
Female	21.5 (2.1‐41.0)	16.9 (8.6‐25.1)		6.3 (0.2‐12.4)	3.4 (1.3‐5.5)		37.9 (24.0‐50.8)	31.1 (21.4‐40.9)	
Housing type			<.001			<.001			<.001
House	100.0 (89.7‐100.0)	90.6 (76.8‐100.0)		19.6 (0.0‐49.3)	49.7 (43.1‐56.3)		84.2 (70.5‐97.9)	83.8 (77.8‐89.7)	
Apartment	0.0 (0.0‐10.3)	1.3 (0.4‐2.3)		20.3 (0.0‐47.6)	25.0 (19.3‐30.6)		2.4 (0.0‐5.3)	2.8 (0.2‐5.5)	
Dormitory	0.0 (0.0‐10.3)	0.0 (0.0‐1.8)		26.2 (0.0‐58.5)	8.7 (5.1‐12.4)		11.0 (0.0‐24.4)	13.4 (7.9‐18.9)	
Community center	0.0 (0.0‐10.3)	0.0 (0.0‐1.8)		0.0 (0.0‐18.5)	1.9 (0.5‐3.4)		0.0 (0.0‐5.0)	0.0 (0.0‐2.0)	
Street or homeless	0.0 (0.0‐10.3)	3.1 (1.3‐5.0)		26.9 (0.0‐54.9)	10.7 (6.5‐14.9)		1.9 (1.0‐2.9)	0.0 (0.0‐2.0)	
Highest level of education completed			<.001			<.001			<.001
None	4.1 (0.0‐9.6)	7.4 (0.0‐22.6)		19.1 (0.0‐43.0)	5.9 (0.1‐11.8)		0.0 (0.0‐5.0)	0.0 (0.0‐2.0)	
Primary	46.4 (25.9‐66.9)	35.1 (24.2‐45.9)		24.1 (0.0‐56.6)	31.8 (20.9‐42.6)		4.0 (0.0‐8.9)	15.8 (9.3‐22.4)	
Secondary	43.0 (24.7‐61.2)	51.8 (39.0‐64.6)		56.9 (23.2‐90.6)	56.3 (44.5‐68.0)		81.9 (70.9‐92.8)	74.9 (65.1‐84.7)	
Tertiary	6.6 (0.0‐13.5)	4.2 (1.8‐6.5)		0.0 (0.0‐18.5)	5.6 (0.0‐14.2)		10.7 (3.3‐18.0)	5.5 (0.0‐13.0)	
Vocational	0.0 (0.0‐10.3)	1.5 (0.4‐2.6)		0.0 (0.0‐18.5)	0.5 (0.0‐1.1)		3.5 (1.4‐5.5)	3.8 (0.2‐7.4)	
Network size^d^			.17			.96			.97
Less than or equal to median	76.3 (65.6‐86.9)	85.1 (81.8‐88.3)		74.0 (48.1‐98.8)	73.8 (69.4‐78.2)		78.1 (70.2‐85.5)	78.3 (73.3‐83.1)	
Greater than median	23.7 (13.1‐34.4)	14.9 (11.7‐18.2)		26.0 (1.2‐51.9)	26.2 (21.8‐30.6)		21.9 (14.5‐29.8)	21.7 (16.9‐26.7)	
Last injected drugs			.27			<.001			.048
Today or yesterday	39.1 (17.9‐60.3)	50.5 (37.9‐63.1)		46.6 (6.6‐86.6)	47.8 (35.9‐59.7)		6.6 (0.7‐12.6)	20.1 (14.0‐26.2)	
In the past week	41.6 (23.6‐59.6)	40.7 (27.6‐53.8)		51.2 (8.8‐93.7)	45.5 (33.5‐57.6)		57.7 (43.1‐71.9)	43.9 (35.5‐52.4)	
In the past month	15.0 (2.4‐27.6)	8.3 (3.6‐12.9)		2.2 (0.0‐6.1)	5.5 (1.6‐9.4)		21.0 (8.9‐33.3)	15.9 (10.3‐21.6)	
In the past 3 months	4.3 (0.0‐9.9)	0.5 (0.0‐1.4)		0.0 (0.0‐18.5)	1.2 (0.0‐6.7)		14.6 (4.2‐25.2)	20.0 (12.7‐27.3)	
Frequency of injection in the last 6 months			<.001			<.001			.98
Less than once a month	0.0 (0.0‐10.3)	1.0 (0.0‐2.4)		0.0 (0.0‐18.5)	2.6 (0.0‐8.2)		15.6 (0.0‐36.4)	11.8 (5.2‐18.5)	
1 to 4 times a month	14.4 (4.7‐24.1)	13.6 (1.6‐25.7)		30.1 (0.0‐71.8)	28.9 (18.0‐39.9)		61.6 (40.4‐82.7)	61.8 (53.5‐70.1)	
2 to 7 times a week/once a day	63.0 (46.6‐79.5)	51.4 (38.9‐63.9)		58.0 (25.9‐90.1)	35.9 (23.5‐48.2)		17.0 (1.6‐32.5)	19.7 (13.8‐25.5)	
2 to 3 times a day	18.0 (5.2‐30.7)	33.3 (24.1‐42.4)		11.9 (0.0‐26.9)	31.8 (23.4‐40.2)		2.9 (0.0‐6.3)	3.1 (1.3‐5.1)	
5 or more times a day	4.7 (2.2‐7.1)	0.7 (0.1‐1.3)		0.0 (0.0‐18.5)	0.8 (0.4‐1.1)		3.0 (0.0‐8.2)	3.5 (1.1‐6.0)	
Used heroin in the last 6 months			.38			.99			.24
Yes	90.0 (82.4‐97.6)	94.5 (90.2‐98.8)		95.9 (92.3‐99.5)	95.9 (93.8‐98.0)		35.4 (20.7‐49.7)	26.5 (18.1‐34.8)	
No	10.0 (2.4‐17.6)	5.5 (1.2‐9.8)		4.1 (0.5‐7.7)	4.1 (2.0‐6.2)		64.6 (50.3‐79.3)	73.5 (65.2‐81.9)	
Locations where self-injected drugs were used in the last 6 months^e^									
At my house	27.2 (12.7‐42.2)	21.2 (15.7‐26.7)	.51	31.2 (8.0‐54.9)	17.9 (12.6‐23.0)	.25	29.2 (17.3‐41.7)	24.7 (16.4‐32.9)	.56
Someone’s house	12.2 (0.0‐24.7)	12.2 (8.3‐16.1)	.99	6.7 (0.0‐31.6)	17.3 (12.5‐22.2)	.32	53.6 (39.4‐67.8)	58.7 (50.2‐67.2)	.54
Street or park	16.5 (3.9‐29.5)	34.4 (27.7‐41.1)	.06	32.6 (0.0‐65.9)	11.2 (6.5‐15.7)	.07	26.3 (14.3‐38.4)	18.6 (11.6‐25.7)	.25
Latrines or public toilets	0.0 (0.0‐10.3)	0.8 (0.4‐1.3)	—^f^	6.8 (0.0‐30.3)	4.7 (2.1‐7.2)	.71	0.0 (0.0‐5.0)	1.3 (0.0‐7.8)	—
At a bar or club	0.0 (0.0‐10.3)	0.6 (0.3‐1.0)	—	0.0 (0.0‐18.5)	3.2 (0.0‐7.0)	—	16.7 (6.4‐26.9)	6.1 (1.9‐10.2)	.04
At the drug dealer’s place	18.4 (5.3‐31.8)	29.0 (23.0‐35.1)	.25	49.9 (21.9‐76.3)	52.5 (46.0‐59.1)	.90	10.1 (1.2‐18.8)	16.3 (10.1‐22.5)	.30
At a shooting gallery	0.0 (0.0‐10.3)	0.0 (0.0‐1.8)	—	28.1 (4.5‐51.4)	22.4 (17.3‐27.6)	.67	1.4 (0.0‐3.3)	0.0 (0.0‐2.0)	—
Other	44.4 (29.6‐58.9)	39.7 (32.2‐47.4)	.67	11.2 (0.0‐25.3)	25.5 (20.1‐30.9)	.18	5.9 (1.2‐10.5)	11.3 (6.8‐15.8)	.20
Frequency of using a new, sterile needle in the last 6 months			.12			<.001			.20
Never	5.0 (1.0‐9.0)	0.9 (0.5‐1.2)		0.0 (0.0‐18.5)	2.8 (0.8‐4.8)		11.8 (2.3‐21.4)	8.2 (4.3‐12.0)	
Rarely	29.4 (13.1‐45.8)	29.9 (17.0‐42.8)		11.7 (0.0‐43.4)	15.6 (11.3‐19.9)		35.0 (22.3‐48.2)	27.5 (19.7‐35.4)	
Half of the time	16.1 (1.4‐30.9)	18.7 (5.8‐31.7)		11.3 (0.0‐36.7)	14.3 (10.3‐18.2)		5.1 (1.3‐9.1)	10.7 (5.9‐15.6)	
Most of the time	5.9 (0.0‐13.7)	19.7 (12.8‐26.7)		18.6 (3.3‐33.9)	15.4 (10.7‐20.2)		25.5 (14.5‐36.1)	22.6 (15.7‐29.4)	
Always	43.5 (22.7‐64.3)	30.8 (19.9‐41.7)		58.4 (27.1‐89.7)	51.9 (45.6‐58.2)		22.6 (11.6‐33.3)	31.0 (23.3‐38.6)	
Frequency of using needles that someone else had already injected with in the last 6 months			.29			<.001			<.001
Never	62.1 (43.6‐80.6)	61.1 (48.4‐73.7)		69.4 (37.0‐100.0)	87.0 (81.9‐92.2)		39.7 (17.9‐61.6)	56.3 (48.1‐64.5)	
Rarely	17.3 (0.0‐34.8)	15.2 (9.8‐20.7)		8.5 (0.0‐35.0)	4.5 (2.4‐6.5)		19.3 (0.0‐39.0)	14.0 (8.4‐19.6)	
Half of the time	2.4 (0.0‐5.2)	13.2 (0.3‐26.1)		22.1 (0.0‐48.8)	6.3 (2.3‐10.3)		12.1 (4.7‐19.4)	7.6 (3.5‐11.7)	
Most of the time	12.0 (5.7‐18.2)	9.1 (4.0‐14.2)		0.0 (0.0‐18.5)	1.2 (0.0‐3.0)		23.0 (8.6‐37.4)	17.6 (11.6‐23.7)	
Always	6.2 (0.0‐13.1)	1.4 (0.0‐3.5)		0.0 (0.0‐18.5)	1.0 (0.0‐2.2)		5.9 (1.8‐10.0)	4.4 (1.5‐7.4)	
Ever injected self with a friend’s blood to get high (blue toothing)			.97			.56			.003
Yes	21.2 (6.7‐35.6)	20.9 (14.8‐27.0)		10.3 (0.0‐40.5)	5.6 (2.2‐9.0)		24.5 (12.8‐36.4)	7.3 (2.8‐11.8)	
No	78.8 (64.4‐93.3)	79.1 (73.0‐85.2)		89.7 (59.5‐100.0)	94.4 (91.0‐97.8)		75.5 (63.6‐87.2)	92.7 (88.2‐97.2)	
Ever been arrested due to injection drug use			.29			.20			.68
Yes	78.7 (67.5‐89.9)	88.6 (83.7‐93.7)		37.8 (9.4‐66.3)	63.3 (57.2‐69.3)		85.8 (68.8‐100.0)	88.4 (83.1‐93.7)	
No	11.4 (3.8‐19.0)	3.7 (0.2‐6.9)		12.6 (0.0‐35.7)	11.2 (7.4‐14.9)		5.3 (0.0‐18.8)	2.5 (0.7‐4.3)	
Ever talked to a peer educator or outreach worker about HIV			.66			.67			<.001
Yes	64.8 (48.5‐81.3)	60.3 (52.5‐67.9)		63.5 (39.1‐88.6)	57.8 (51.3‐64.3)		65.2 (52.1‐78.7)	55.8 (47.7‐63.8)	
No	35.2 (18.7‐51.5)	39.7 (32.1‐47.5)		36.5 (11.4‐60.9)	42.2 (35.7‐48.7)		34.8 (21.3‐47.9)	44.2 (36.2‐52.3)	
Ever tested for HIV			.18			.98			
Yes	97.2 (92.0‐100.0)	89.2 (84.1‐94.3)		79.5 (55.3‐100.0)	79.7 (74.5‐84.8)		91.0 (77.5‐100.0)	85.8 (79.8‐91.8)	.40
No	2.8 (0.0‐8.0)	10.8 (5.7‐15.9)		20.5 (0.0‐44.7)	20.3 (15.2‐25.5)		9.0 (0.0‐22.5)	14.2 (8.2‐20.2)	
Ever received drug treatment			<.001			.72			.91
Yes	0.0 (0.0‐10.3)	5.1 (1.7‐8.5)		7.0 (0.0‐31.1)	9.7 (6.2‐13.3)		5.8 (3.0‐8.7)	6.3 (2.2‐10.4)	
No	100.0 (89.7‐100.0)	94.9 (91.5‐98.3)		93.0 (68.9‐100.0)	90.3 (86.7‐93.8)		94.2 (91.3‐97.0)	93.7 (89.6‐97.8)	

a Previously captured represents people who inject drugs who participated in the biobehavioral survey (capture 3) and reported receiving an object in captures 1 or 2.

b CI: confidence interval.

c *P* values computed using Kruskal test for difference in medians, and a survey-weighted quasi-binomial general linear model for categorical variables.

d Median network size was 5, 4.5, and 3 in Livingstone, Lusaka, and Ndola, respectively.

e Responses not mutually exclusive.

f Indicates that *P* values were not calculated due to insufficient data or no variation between groups.

### SS-PSE

The SS-PSE was highest in Lusaka (4350, 95% CI 1410‐18,890), followed by Ndola (2010, 95% CI 690‐7400) and Livingstone (450, 95% CI 340‐810; Table 2). For all cities, SS-PSE was higher without imputed visibility than with it. SS-PSE with imputed visibility was 360 (95% CI 290‐530) in Livingstone, 2040 (95% CI 660‐30,410) in Lusaka, and 760 (95% CI 390‐3060) in Ndola.

### Bayesian Consensus PSE

Integrating 3S-CRC and SS-PSE with imputed visibility estimates, the final Bayesian consensus PSE ranged between 0.5% to 1.8% of the adult male population and all below 1% of the total adult population in each city (Table 4). The consensus PSE was highest in Lusaka (3700, 95% CRI 1500‐7500), followed by Ndola (2200, 95% CRI 1600‐2900), and Livingstone (1200, 95% CRI 900‐1900).

**Table 4.** Bayesian consensus population size estimates for people who inject drugs by city, Zambia, 2021.

City	Estimate (median)	95% CRI^a^	% of males aged 15‐64 years district population^b^^,^^c^	% of total people aged 15‐64 years district population^b^^,^^c^
Livingstone	1200	900‐1900	1.8 (1.4‐2.9)	0.9 (0.7‐1.5)
Lusaka	3700	1500‐7500	0.5 (0.2‐0.9)	0.2 (0.1‐0.5)
Ndola	2200	1600‐2900	1.1 (0.8‐1.5)	0.6 (0.4‐0.7)

a CRI: credible interval.

b District population estimates were used because estimates for smaller catchment areas were not available. In addition to the estimated percentage of the total district population, the estimated percentage of males in the district population is provided because site samples have a high proportion of men. Estimates and CRIs are rounded to the nearest 50. Further, 95% CRI indicate the interval where the true population parameter falls with 95% probability, given the observed data.

c District population [23].

## Discussion

### Principal Findings

Our study found higher estimates of people who inject drugs than prior estimates of people who inject drugs in other parts of Zambia [7], yet ours were relatively consistent with estimates for sub-Saharan Africa where prevalence of injection drug use is estimated to be 0.3% (0.1%‐0.6%) of the adult population and 0.5% (0.1%‐1.0%) among adult males [3]. The prior PSE study in Zambia estimated a much lower number than our Bayesian consensus estimates and provided no uncertainty ranges; a total of 907 people who inject drugs across all 3 districts were estimated after applying correction factors for census enumeration, and 846 people who inject drugs were estimated using wisdom of the crowd methods [7]. The methods used in the prior PSE study in Zambia had several limitations. It applied census methods to enumerate the number of people who inject drugs at hotspots and used wisdom of the crowd methods with purposively recruited “gatekeepers” in Solwezi, Kitwe, and Kabwe [7]. Both methods likely underestimated the number of people who inject drugs, given biases in location sampling.

3S-CRC, used in our study, has increasingly been used to estimate the population size of key populations [24-30] and has several strengths. Estimation using 3 or more captures can account for heterogeneity among captures, including subpopulations with different behaviors or social visibility. In our study, there were more recaptures between location-based captures (2S-CRC) than with the RDS survey (3S-CRC), suggesting that the RDS survey was able to reach people who were less likely to be found in unique object distribution locations. Future research should explore heterogeneity within networks for people who inject drugs and how differences in recruitment methods influence both recruitment probabilities and the resulting PSE.

BBS participants who had been captured in capture 1 or 2 had differences in housing type and level of education compared to BBS participants not previously captured in all cities, though variations of results by city were seen. These 2 groups had differences in the frequency of sharing needles and frequency of injection, time of last injection, experiences with peer educators or outreach workers, and receipt of drug treatment in one or more cities. Notably, heterogeneity in results by city suggest biases in recruitment methods may be more localized. For example, in Ndola, BBS participants not previously captured via location sampling had lower engagement with a peer educator or outreach worker than those who were previously captured, while other cities had no differences in engagement between these groups.

It is important to acknowledge the assumptions of the PSE approaches used in this study. SS-PSE relies on prior knowledge or assumptions about the target population and assumes a fully connected social network among the target population; given this was the first BBS among people who inject drugs in Zambia, inputs used to generate PSEs through SS-PSE relied on information from prior studies in other settings and may not accurately reflect the context for these geographies in Zambia. Similar assumptions about a connected social network apply to the RDS recruitment process, including the validity of the sample as the third capture-recapture group. Capture-recapture methods ideally assume a closed population, independent capture samples, and equal probability of capturing individuals in the target population, though the use of 3 or more captures and appropriate estimators can allow for some relaxation of these assumptions.

Efforts to minimize the violation of these assumptions were taken in this study. To minimize in- or out-migration of people who inject drugs, the first 2 captures occurred over a short duration, and all captures occurred no more than a week apart. People who inject drugs were sampled from a different set of locations in captures 1 and 2 to help ensure independence and seeds for RDS were selected independently from unique object distributors.

### Limitations

We were not able to produce separate PSEs for people who inject drugs by sex, as data on sex were not collected in captures 1 or 2. While we had planned to produce PSEs using service multiplier methods, it was not possible due to limited service providers and a lack of people who inject drugs–specific program data in Zambia. We used wisdom of the crowd methods via the RDS survey, where BBS participants were asked to provide their best estimate (or “most accurate” estimate), as well as their estimate of the highest and lowest possible number of people who inject drugs, aged 16 years or older, living in the survey city. Ultimately, given concerns about the quality and weakness of the method, PSEs generated using wisdom of the crowd were omitted from the Bayesian consensus estimate and excluded from this paper. Some activities in this study were implemented during restrictions associated with the COVID-19 epidemic, which may have limited access to subpopulations of people who inject drugs, particularly those who may have been reached via location sampling methods.

### Conclusions

Using rigorous methods, this study provides PSEs for people who inject drugs in Zambia and is the first to produce PSEs for major geographies in the country, including Lusaka, Livingstone, and Ndola. We use multiple methods to derive PSEs; present findings, strengths, and limitations of each method; and document our approach to generating Bayesian consensus estimates. Findings from this study highlight heterogeneity among people who inject drugs in Zambia, including that some people who inject drugs may not be accessible via location sampling, which can guide programming and surveillance strategies to reach these groups. Transparency around underlying data and methodological approaches used to generate consensus estimates, as provided here, can support the field and enhance modeling activities, including extrapolation of size estimation. Critically, the consensus PSE generated in this study can inform HIV and HBV/HCV prevention and treatment program planning, including target setting and coverage estimation, and guide future surveillance activities for key populations in the country.

## Abbreviations

2S-CRC: 2-source capture-recapture
3S-CRC: 3-source capture-recapture
BBS: biobehavioral survey
CI: confidence interval
CRI: credible interval
HBV: hepatitis B virus
HCV: hepatitis C virus
PSE: population size estimate
RDS: respondent-driven sampling
SS-PSE: successive sampling population size estimation
